# Fuzzy Controller Implemented for Movement of a Tendon-Driven 3D Robotic Lumbar Spine Mechanism †

**DOI:** 10.3390/s23249633

**Published:** 2023-12-05

**Authors:** Thuanne Paixão, Ana Beatriz Alvarez, Ruben Florez, Facundo Palomino-Quispe

**Affiliations:** 1 PAVIC Laboratory, University of Acre (UFAC), Rio Branco 69915-900, Brazil; ana.alvarez@ufac.br; 2 LIECAR Laboratory, Universidad Nacional de San Antonio Abad del Cusco (UNSAAC), Cuzco 08003, Peru; rubendfz2206@gmail.com (R.F.); facundo.palomino@unsaac.edu.pe (F.P.-Q.)

**Keywords:** biomechanics, robotic lumbar spine, fuzzy control, Mamdani fuzzy logic

## Abstract

Notable efforts have been devoted to the development of biomechanical models of the spine, so the development of a motion system to control the spine becomes expressively relevant. This paper presents a fuzzy controller to manipulate the movement of a 3D robotic mechanism of the lumbar spine, which is driven by tendons. The controller was implemented in Matlab/Simulink R2023a software, MathWorks (Brazil), considering mathematical modeling based on the Lagrangian methodology for simulating the behavior of the lumbar spine dynamic movement. The fuzzy controller was implemented to perform movements of two joints of the 3D robotic mechanism, which consists of five vertebrae grouped into two sets, G1 and G2. The mechanism’s movements are carried out by four servomotors which are driven by readings from two sensors. For control, the linguistic variables of position, velocity and acceleration were used as controller inputs and the torque variables were used for the controller output. The experimental tests were carried out by running the fuzzy controller directly on the 3D physical model (external to the simulation environment) to represent flexion and extension movements analogous to human movements.

## 1. Introduction

The construction of biomechanical models in relation to the structural and functional analysis of the behavior of elements that represent specific characteristics of human biological anatomy has become extremely relevant for the development of research and production of robotic mechanisms capable of replicating physical constitutions with kinesiological aspects of the movement of human body segments. From this perspective, many research projects focus on designing specific models of anatomical structures, highlighting the inherent physiological properties of regions that are arranged in the various parts of the human body [1,2]. Biomechanical models are also needed to analyze human posture for rehabilitation purposes [3]. In view of this, the development of 3D models of the human spine results in several benefits, considering the fact that it is a vital region for the arrangement of balance and dynamic stability of movement.

The pathological dysfunctions that occur in the lumbar spine as a result of external factors related to sedentary lifestyles and poor posture, among others, and which cause injuries and degeneration of this structure, impair the quality of life of human beings [4]. There are notable efforts dedicated to the composition of biomechanical models that represent this region of the spine. These representations can be based on 3D prints or other technologies that can simulate the morphological composition of the vertebrae and spine as well as their anatomical characteristics, making it possible to study and analyze the kinematic and dynamic properties of these elements in relation to their behavior and physical arrangement [5,6].

In order to make these representations genuine and effective, the development of a movement system for these biomechanical structures needs to be designed that can realize movements analogous to those of the human body. As a computational intelligence control strategy, the fuzzy controller has important properties that can be applied to complex systems that exhibit imprecise behavior. This intelligent control technique has been widely used to eliminate noise and promote control efficiency for autonomous robotic systems [7]. In this way, the fuzzy controller becomes an exceptional option for controlling the movement of biomechanical structures, where linguistic terms that are normally only used in natural human language can be inserted, simulating the human ability to estimate predictions in situations of uncertainty through subjective or approximate concepts and a set of rules, which in many cases can be used to replace complex mathematical models [8].

The construction and validation of an experimental kinesiological model of the human lumbar spine was developed by Angst [9] with the main focus on the anatomical musculoskeletal arrangement of movement in relation to the flexion and extension of the constructed structure. The 3D biomechanical model consisted of the design of the lumbar segment and the sacrum, which are present in the structure of the human spine. The authors of this paper, at [10], developed the lumbar spine model, which was based on Lagrange’s methodology, with two DOFs (Degrees of Freedom) for manipulating the movement of two vertebrae of the lumbar spine. The model’s behavior was simulated with Matlab/Simulink software using a conventional PID (Proportional Integral Derivative) controller. Next, in [11], the main features of the development of a 3D model of the lumbar spine were presented, built and printed using 3D Slicer 4.11, SolidWorks 2022 and Ultimaker Cura 4.5 software (Brazil). The physical implementation of motion control was composed for a simplified mechanism of the 3D structure, using nylon propulsion tendons and electronic devices to reproduce flexion and extension movements.

This paper present a fuzzy controller for the movement of a tendon-driven robotic model of the lumbar spine. A fuzzy controller was implemented to control the movement of two vertebras of the robotic structure, providing control of the lumbar spine model. The robotic structure uses the 3D model of the lumbar spine proposed by Angst [9]. The variables position, speed and acceleration constituted the inputs for the fuzzy controller based on rules and specific membership functions. The performance results of the fuzzy controller were verified after implementing the control system in the robotic structure based on the 3D model of the lumbar spine. In this way, the fuzzy controller started to act directly on the physical model (external to the simulation environment), and the experimental tests were carried out with the execution of flexion and extension analogous to human movements.

## 2. Related Work

In the literature, some works can be found reporting on the construction and manipulation of structures with biomechanical characteristics. The development of a spinal mechanism for humanoid robots described in the work by Kakehashi et al. [12] seeks to simulate the human musculoskeletal structure, reproducing the movements of flexion, lateral flexion, rotation and extension, with the drive of nine motors from tendons for the cervical, thoracic and lumbar segments. Similarly, in the work by Clifton et al. [13], a three-dimensional model consisting of multiple thermoplastic polymers, with spongy cortical characteristics of the vertebrae, was built by 3D experimental printing of lumbar and cervical vertebrae with the aim of providing the use of 3D models for simulations of spinal surgeries.

In the work by Bohl et al. [14] describe the development of a synthetic biomechanical model of the spine in relation to the range of movement and the similarity of the developed material to human tissue. The tested movements of flexion, extension, lateral bending, rotation and axial compression were compared with previous cadaveric control data. In another paper by Bohl et al. [15], validation tests were carried out on a 3D-printed vertebral body of the lumbar spine with regard to torque tests, axial pull-out force and material stiffness with the aim of evaluating the biomechanical behavior of the printed vertebral body in relation to characteristics of cadaveric human bones. In the work by DiAngelo et al. [16], a synthetic model of the lumbar spine consisting of the vertebral body T12 to the sacrum was used for specific performance analyses of flexion and extension stiffness as well as segment and global kinematic properties. The tests were carried out on a robotic platform, and the test responses were compared with in vivo and in vitro studies.

On the other hand, there are studies that show the efficiency of 3D-printing technologies, such as the work by Li et al. [17] where an analysis was made of the effectiveness of using 3D-printed lumbar spinal models to improve preoperative surgical planning in revisions of lumbar discectomy surgeries, highlighting the application of these models also for educational and demonstration purposes for patients. In the same vein, a systematic review of work carried out using three-dimensional printing (3DP) in relation to spinal surgical procedures was carried out by Wilcox et al. [18] with a focus on the application of 3D printing of spinal models in planning, trials and implants using this technique. Likewise, in the work by Cho et al. [19], a review of the literature on 3D printing of the spine for preoperative planning and surgical simulations was carried out with the aim of emphasizing the application of the functionality of this technology for the education of residents and patients as well as the main limitations and future developments.

With a view to robust control of mechanisms, some studies have developed fuzzy controllers for manipulating robotic systems. In the work by Urrea et al. [20], a fuzzy control proposal is presented for a two DOFs (Degrees of Freedom) manipulator robot, where the linguistic variables of position, velocity and acceleration were inserted into the controller structure and implemented for each joint of the manipulator robot. Simulation results in Matlab/Simulink are presented. Similarly, in the work by Bikova et al. [21], fuzzy controllers were developed and simulated for tracking the trajectory of a robot with two DOFs as a proposal for use in cardiac surgery manipulators, where the linguistic variables of position, velocity and acceleration were also applied to each joint of the robot.

Based on the authors’ previous work and the references, this paper presents the development of a fuzzy controller for the movement of a robotic lumbar spine structure. Unlike the works found in the literature, which take place in a simulation environment, this paper presents the fuzzy controller implemented in the 3D model [9] following the physical scheme for implementing the control devices according to the description set out in [11]. Inherent results of the physical behavior of the controlled movement of the 3D robotic biomechanical structure driven by tendons are presented.

## 3. Implementation of the Motion Control System

### 3.1. Mathematical Modeling of the Dynamic Model

**Figure 1:**
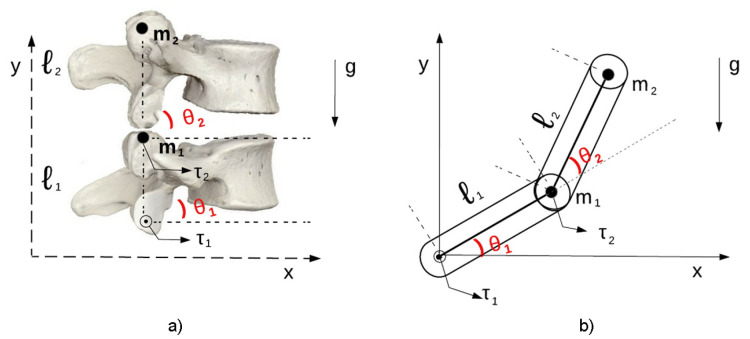
Model used for simulation [10]: (**a**) dynamic control model 2 DOFs. (**b**) Diagram of a 2 DOFs robotic manipulator.

Based on [10], the mathematical modeling proposed for a robotic structure of a two DOFs manipulator was used to represent the dynamic structure of the human lumbar spine (movement of two vertebrae). Figure 1 shows the analogies between the manipulator’s movement and its specific characteristics, where $m_{1}$ and $m_{2}$ refer to the masses of the vertebrae, $l_{1}$ and $l_{2}$ refer to the heights of each vertebra, $\theta_{1}$ and $\theta_{2}$ refer to the movement angles, *g* refers to the gravitational force, and $\tau_{1}$ and $\tau_{2}$ refer to the respective torques, which generate the forces applied to the joints of each vertebra.

This was used to establish the dynamic requirements needed to build the mathematical model. Based on the formulations of the dynamic model for the robotic manipulator, the representation based on the Lagrangian methodology was constructed as shown in Equation (1).

(1) $$\tau =M\left(q\right)\ddot{q}+C\left(q,\dot{q}\right)+G\left(q\right)$$

where M(q) refers to the inertia matrix, $C(q,\dot{q})$ represents the coriolis/centrifugal force, G(q) presents the gravity force of the vector, *q* characterizes the position, $\dot{q}$ refers to the velocity and $\ddot{q}$ comprises the acceleration. For the simulation of the model in the computational tool, we considered the matrix representation of the model whose components are described in Equations (2)–(6), which show the composition of the inertia matrix, Equations (7)–(9), which refer to the vector of coriolis/centrifugal forces and Equations (10)–(12), which identify the vector of gravity torques.

(2) $$M=\left[\begin{array}{cc} M_{11} & M_{12}\\ M_{21} & M_{22} \end{array}\right]$$

(3) $$M_{11}=\left(m_{1}+m_{2}\right)l_{1}^{2}+m_{2}l_{2}^{2}+2m_{2}l_{1}l_{2}\cos\theta_{2}$$

(4) $$M_{12}=m_{2}l_{2}^{2}+m_{2}l_{1}l_{2}\cos\theta_{2}$$

(5) $$M_{21}=m_{2}l_{2}^{2}+m_{2}l_{1}l_{2}\cos\theta_{2}$$

(6) $$M_{22}=m_{2}l_{2}^{2}$$

(7) $$C=\left[\begin{array}{c} C_{11}\\ C_{21} \end{array}\right]$$

(8) $$C_{11}=-m_{2}l_{1}l_{2}\left(2{\dot{\theta }}_{1}{\dot{\theta }}_{2}+{\dot{\theta }}_{1}^{2}\right)\sin\left(\theta_{2}\right)$$

(9) $$C_{21}=-m_{2}l_{1}l_{2}{\dot{\theta }}_{1}{\dot{\theta }}_{2}\sin\left(\theta_{2}\right)$$

(10) $$G=\left[\begin{array}{c} G_{11}\\ G_{21} \end{array}\right]$$

(11) $$G_{11}=-\left(m_{1}+m_{2}\right)gl_{1}\sin\left(\theta_{1}\right)-m_{2}gl_{2}\sin\left(\theta_{1}+\theta_{2}\right)$$

(12) $$G_{21}=-m_{2}gl_{2}\sin\left(\theta_{1}+\theta_{2}\right)$$

These characteristics model the applied functions of the robotic structure, where the values of the $\theta_{1}$ and $\theta_{2}$ angles are established as identifiers of the structure’s output so that they can then be used to feed back the system’s controllers.

### 3.2. Development of the Fuzzy Controller

**Figure 2:**
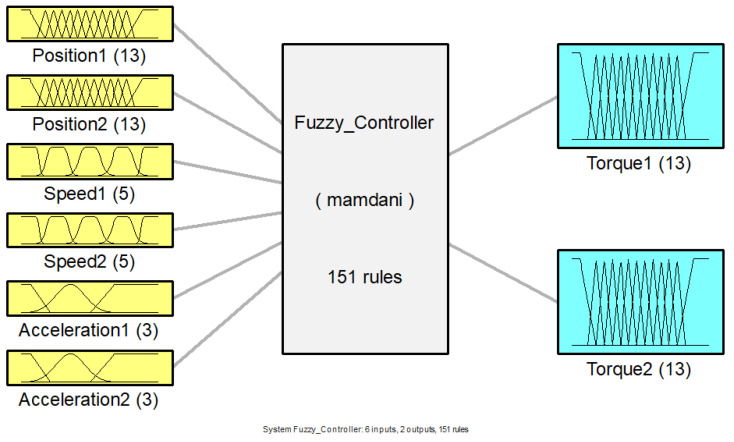
Fuzzy controller composition structure.

The developed fuzzy controller was designed following some characteristics presented in the works of Urrea et al. [20] and Bikova et al. [21]. This controller comprised six input variables, referring to angular position, speed and acceleration and two output variables indicating Torque 1 and Torque 2; in addition, 151 inference rules were implemented for this mechanism, and the composition of the fuzzy controller is exposed in Figure 2.

**Figure 3:**
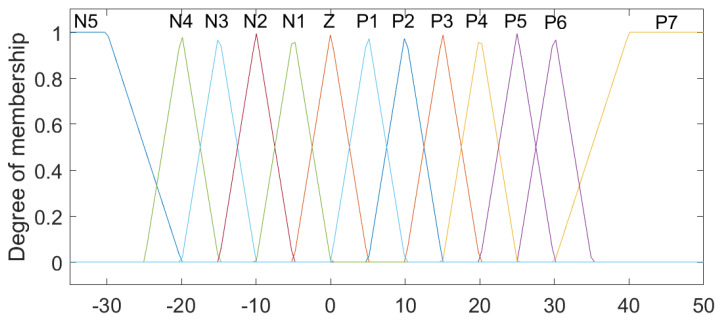
Fuzzy controller association functions for Position 1 and Position 2.

**Figure 4:**
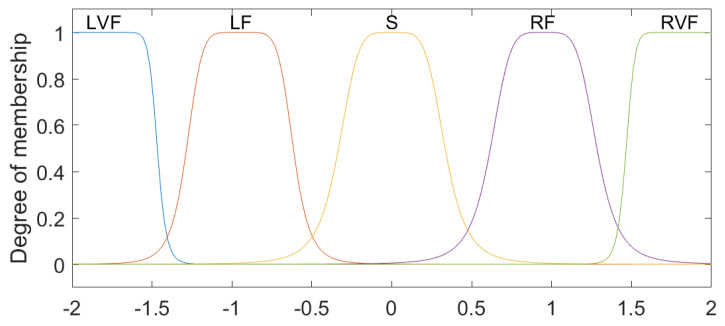
Fuzzy controller association functions for Speed 1 and Speed 2.

**Figure 5:**
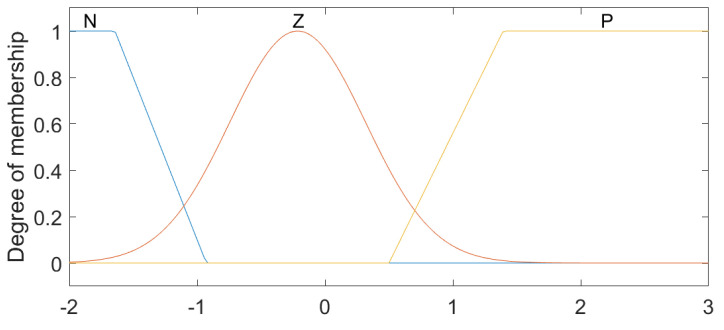
Fuzzy controller association functions for Acceleration 1.

**Figure 6:**
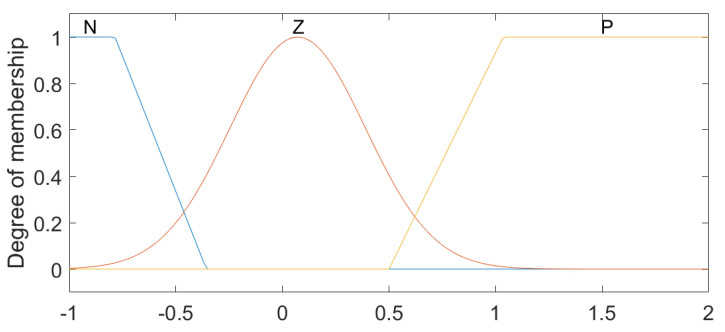
Fuzzy controller association functions for Acceleration 2.

**Figure 7:**
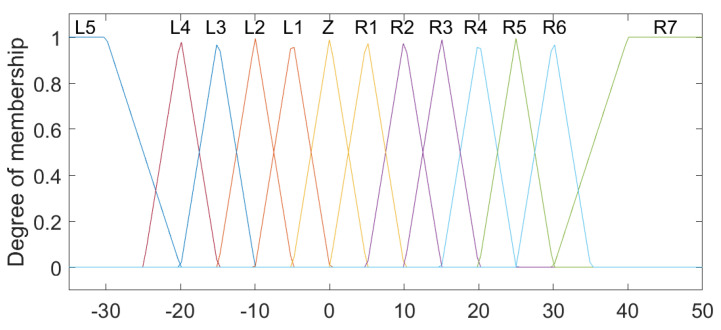
Fuzzy controller association functions for Torque 1 and Torque 2.

The linguistic variables for Position 1 and Position 2 were defined based on the physical characteristics of the lumbar spine [9], with the ranges from −30 to 50, with the linguistic values (N1–5), Z and (P1–7), which represent levels (Negative from 1 to 5), Zero and (Positive from 1 to 7), respectively (Figure 3). For the variables established as Speed 1 and Speed 2, the intervals were classified between the values −2 and 2, comprising the states LVF, LF, S, RF and RVF, which are characterized as Left Very Fast, Left Fast, Slow, Right Fast and Right Very Fast (Figure 4). Regarding the linguistic variables of Acceleration 1 and Acceleration 2, the ranges were determined as −2 to 3 for Acceleration 1 and −1 to 2 for Acceleration 2 with the linguistic states N, Z and P, which are equivalent to Negative, Zero and Positive (Figure 5 and Figure 6). The controller output variables represented as Torque 1 and Torque 2 comprised the ranges from −30 to 50 with the linguistic states (L1–5), Z and (R1–7), representing levels (Left from 1 to 5), Zero, and (Right from 1 to 7), respectively (Figure 7).

The rules for the fuzzy controller were established according to the “If–Then” type. An example of the logical condition of the rule base is described in (13).

(13) $$\begin{array}{c} \mathrm{If}\;Position\;1\;is\;N5\;and\;Position\;2\;is\;N2\;and\;Speed\;1\;is\;LVF\;and\;Speed\;2\;is\;LVF\\ Then\;Torque\;1\;is\;L4\;and\;Torque\;2\;is\;L3. \end{array}$$

In Table 1 and Table 2, the associations of rules implemented for the proposed fuzzy controller are presented, based on logical conditions, through the inference mechanism. Given this, specific rules were established for the relationship between Position 1 and Position 2 with Speed 1 and Speed 2 as well as Position 1 and Position 2 with Acceleration 1 and Acceleration 2 in order to correlate with the output variables referring to Torque 1 and Torque 2.

The method used for the defuzzication stage of this fuzzy controller refers to the approach established for the center of the area (centroid). In this context, this controller follows the Mamdani-based inference methodology.

### 3.3. Fuzzy Controller Implementation

**Figure 8:**
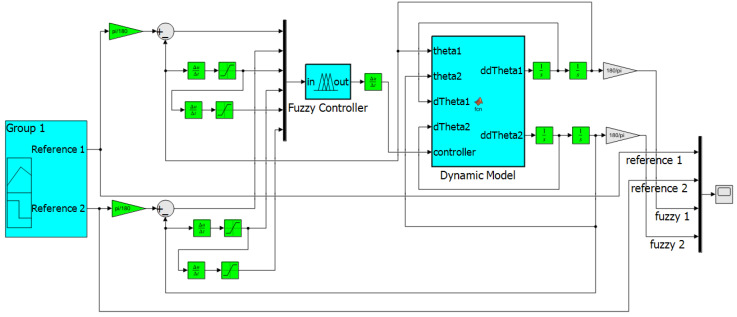
Control system for a robotic manipulator 2 DOFs.

The system for the composition of the movement control of the lumbar spinal robotic model was built using the Matlab/Simulink^®^ software and can be viewed in Figure 8. The dynamic model based on the Lagrange approach, proposed for the composition of the Dynamic Model block, is described in Section 3.1 of this paper. The execution of the control process occurred in accordance with the operating methodology of the closed-loop control system, where the control process receives power through the error, which represents the difference between the reference signal indicated at the system input and the output signal of the controlled process.

The fuzzy controller reference inputs, referring to position, were established separately. The other controller inputs receive signals that are sent for derivative treatments before entering the fuzzy controller. In this way, the fuzzy controller receives Position 1 as its first input, referring to reference signal 1, the third input stipulated for the controller refers to Speed 1, which represents the first derivative of the position in relation to time, and the fifth input of the fuzzy controller consists of Acceleration 1, which characterizes the second derivative of the position in relation to time. This same process occurs for the reference 2 input signal, for Position 2, Speed 2 and Acceleration 2. The fuzzy controller generates the Torque 1 and Torque 2 signal at the output; in this case, these signals are intended for the control block of the dynamic model of the robotic structure.

Based on the block diagram in Simulink, the dynamic model block is characterized by the establishment of the manipulation of received signals for the conception of the proposed degrees of freedom where the characteristics of the model are evaluated and the references inserted in the system are identified with relation to the behavior of the movement signal. In this sense, the six inputs are referring to $\theta_{1}$ and $\theta_{2}$, the first derivative of $\theta_{1}$ and the first derivative of $\theta_{2}$, and the outputs of the fuzzy controller. The structure of the movement control comprises the output of the dynamic block, the second derivative of $\theta_{1}$ and the second derivative of $\theta_{2}$.

## 4. Physical Experiments

### 4.1. Motion Control in the 3D Robotic Lumbar Spine Mechanism

To carry out the physical experiments, two sensors (MPU6050) remained positioned on the L1 and L4 vertebrae of the 3D lumbar spine model, and two groups were established to reproduce 2 degrees of freedom, according to the mathematical modeling proposed for the system. In these groups, the five grouped vertebrae were considered, L1–L2–L3 forming the G1 group and L4–L5 forming the G2 group, as described in [11].

**Figure 9:**
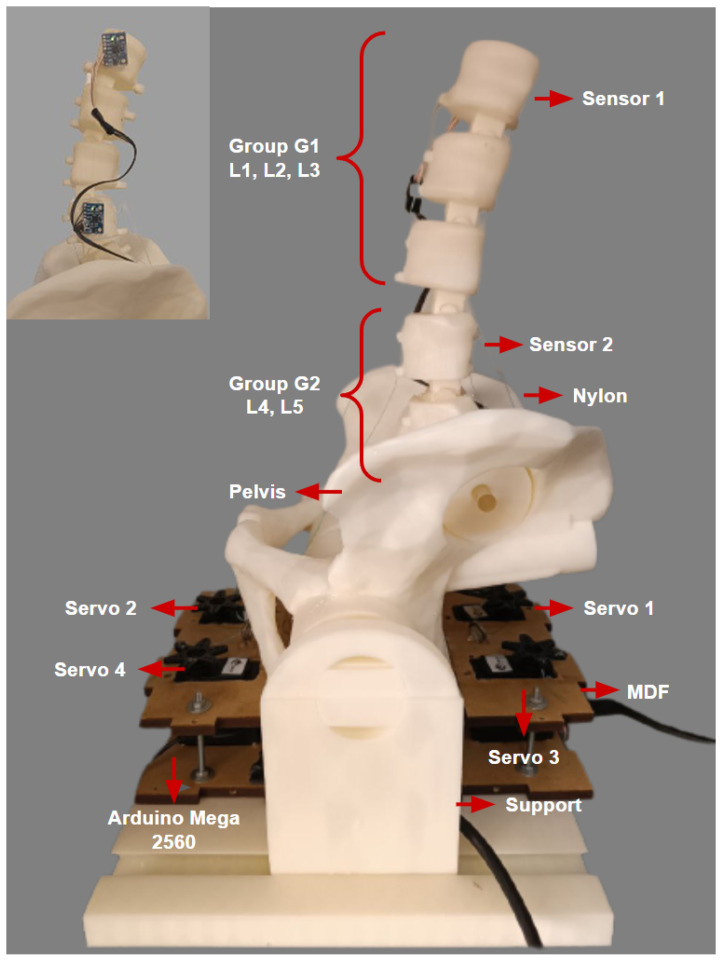
Physical implementation of electronic devices in the 3D model of the lumbar spine. In detail, the physical layout of the sensors.

The mathematical modeling and proposed controller were developed to act based on this 3D model of the lumbar spine, where it became possible to implement the movement control. The complete physical implementation of the 3D robotic lumbar spine model can be seen in Figure 9. The main elements that make up the robotic structure consisted of the five lumbar vertebrae, pelvis, model support, MDF board (Medium-Density Fiber), four MG995 servomotors, two MPU6050 sensors, one Arduino Mega with ATmega2560 controller, one power supply 12 V to 1.5 A with DC-DC LM2596 step-down 3 A voltage converter, and nylon wires. The Arduino Mega implements the specifications for the input and output sets and the rule base for the fuzzy controller.

From the two output signals of the fuzzy controller described in the previous section, representing the torque, the four servomotors were activated through the nylon wires, which represent the structure’s tendons, to perform the flexion and extension movements of the 3D model of the lumbar spine. With torque output signal 1, for extension movements, Servo 1 follows the output angle equivalent to $\theta_{1}$ (Group G1) and Servo 2 supports the movement also following $\theta_{1}$. Considering the bending movements, Servo 2 follows the exit angle related to $\theta_{1}$ (Group G1) and Servo 1 complements the movement also following $\theta_{1}$. In relation to the output signal for Torque 2, the same servomotor activation process occurs; in this case, Servo 3 reproduces the behavior of $\theta_{2}$ (Group G2) for the extension displacement and Servo 4 assists. For the flexion movement, Servo 4 follows the angle established by $\theta_{2}$ and Servo 3 completes the movement.

### 4.2. Positions for Experimental Testing

**Figure 10:**
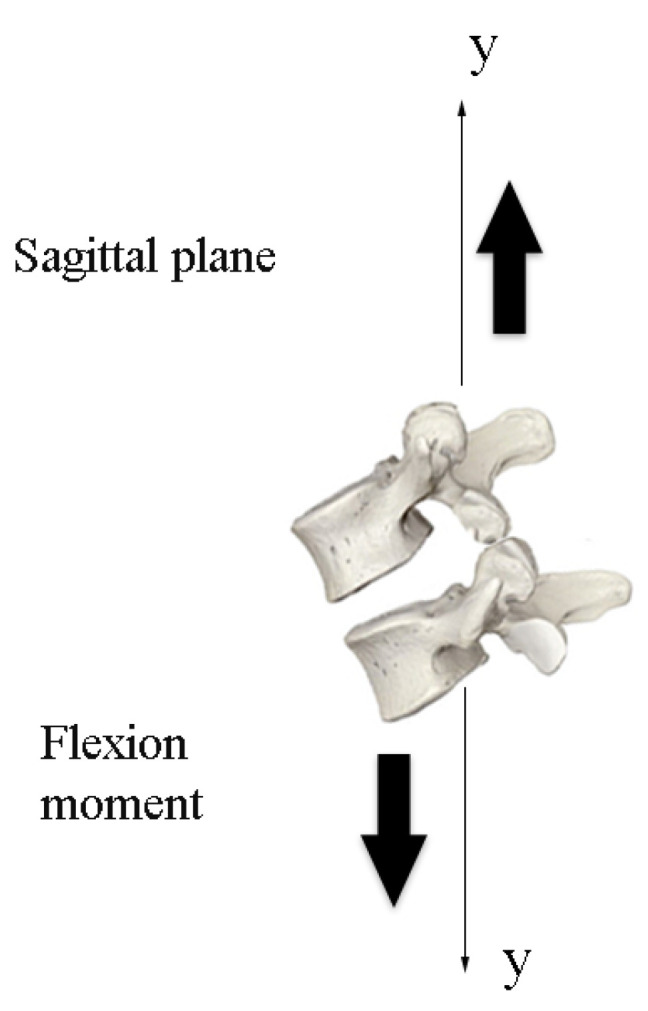
Plane and direction of flexion movement.

**Figure 11:**
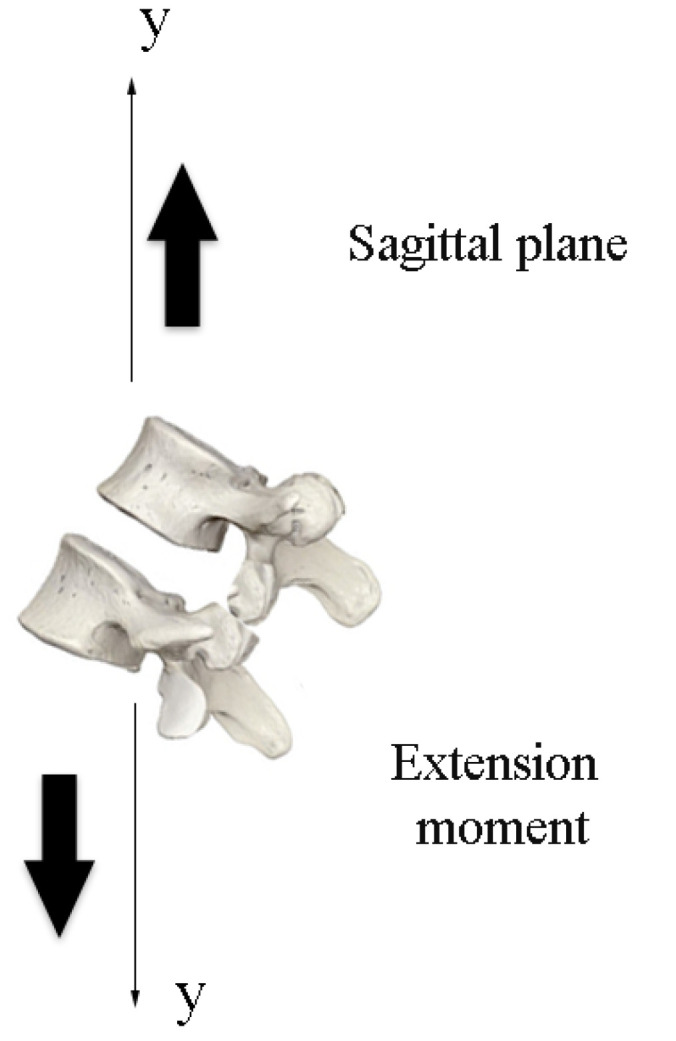
Plane and direction of extension movement.

The initial position of the 3D model of the robotic lumbar spine was established based on the work of Hey et al. [22], who carried out a study with the aim of classifying the posture of the regular alignment of the spine in relation to the orientation of the sagittal plane in a group of 169 adults, in which the inclinations and deviations of the vertebrae of the spine of healthy adults were observed using radiographs, using the types of Roussouly curves as a verification parameter. The positions for the intended flexion and extension movements were determined based on the work of Mitchell et al. [23], who carried out an assessment of the posture of the lumbar spine of 160 students to demonstrate the changes in the alignment of the lumbar spine in relation to low back pain. In this sense, the posture of the lumbar spine of healthy students and students with lower back pain were compared for different ranges of movement. Therefore, the movements for the tests with the 3D lumbar spine model were set at −5° extension and +4° flexion for the initial movement, −35° extension and −10° extension for the extension movement, and +50° flexion and +10° flexion for the flexion movement. Considering the *y*-axis of the central coordinate system, Figure 10 and Figure 11 show the sagittal plane and direction of movement for flexion and extension, respectively.

### 4.3. Experimental Results with Fuzzy Controller

**Figure 12:**
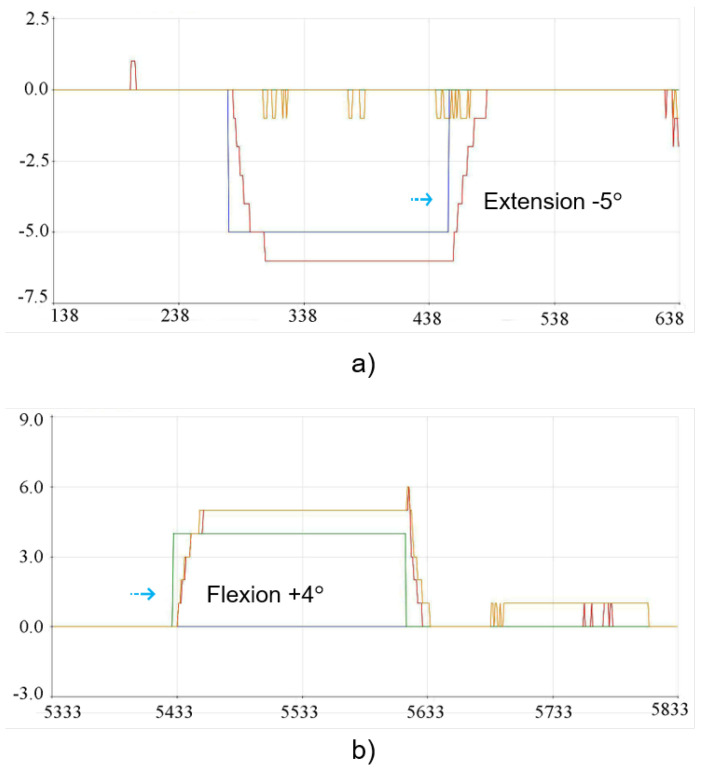
Implementation results for the initial position: (**a**) Extension movement −5° for G1. (**b**) Flexion movement +4° for G2.

**Figure 13:**
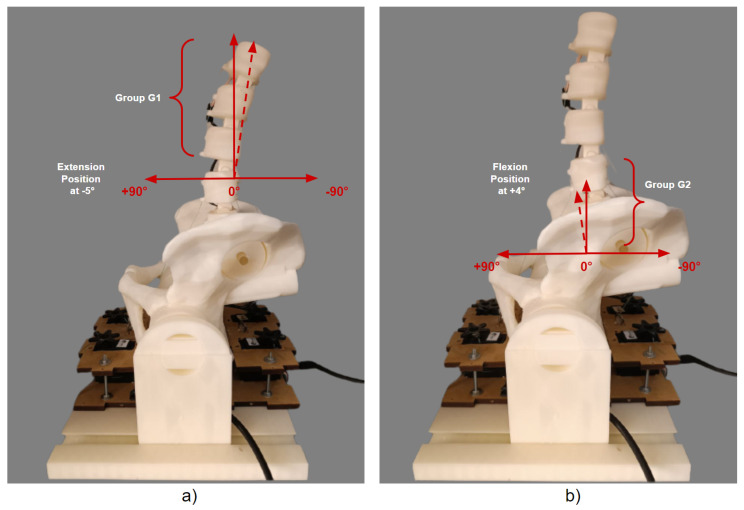
Individual physical results of the starting position for G1 and G2: (**a**) Individual movement of −5° for G1. (**b**) Individual movement of +4° for G2.

In the experimental physical tests carried out with the 3D lumbar spine model, the reference positions for group G1 (L1, L2, L3) were represented by $\theta_{1}$ and the positions defined for group G2 (L4, L5) corresponded to $\theta_{2}$. The tests were carried out in two different stages, where the first stage consisted of indicating an individual position for G1, allowing the movement to be carried out and then returning to the starting position and then carrying out the individual movement for G2, following the same criteria. On the other hand, in the second stage of the tests, a position was indicated for G1, and after the movement had been carried out, this desired position was maintained in order to carry out the intended movement for the G2 group. In this way, individual movements were initially determined for G1 and G2, in which case it became possible to check the behavior and disposition of each group in isolation. The graphs shown in Figure 12 show the performance of the control system running through the implementations carried out. In order to represent the initial position in the 3D structure, it was necessary to indicate −5° for extension for G1 and +4° for flexion for G2. The physical positioning after each individual movement related to the initial position for the 3D model can be seen in Figure 13.

**Figure 14:**
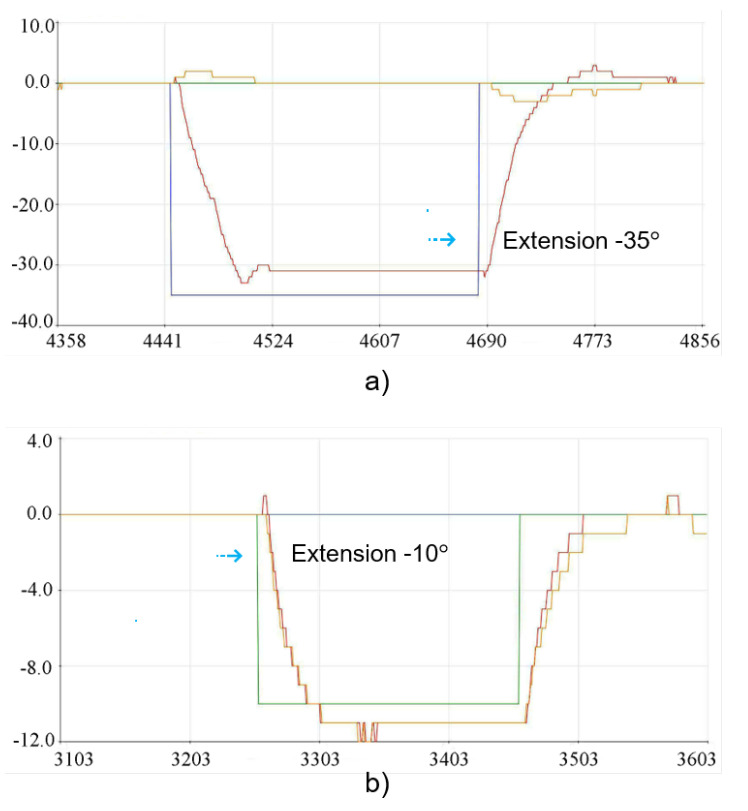
Implementation results for the extension position: (**a**) Extension movement −35° for G1. (**b**) Extension movement −10° for G2.

**Figure 15:**
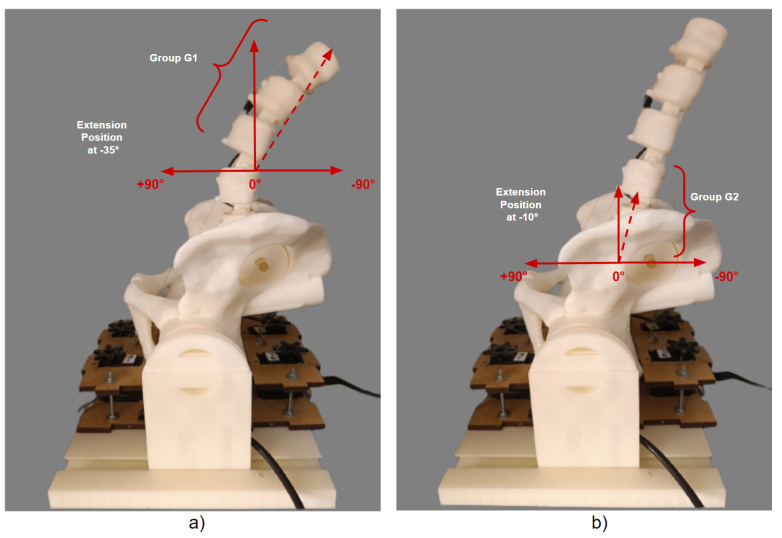
Individual physical results of the extension position for G1 and G2: (**a**) Individual movement of −35° for G1. (**b**) Individual movement of −10° for G2.

The implementation movements for the −35° extension position for G1 and −10° for G2 can be seen in the graphs in Figure 14. Similarly, the tests were carried out individually, and the result of the physical positioning after the movements performed on the 3D model can be seen in Figure 15.

**Figure 16:**
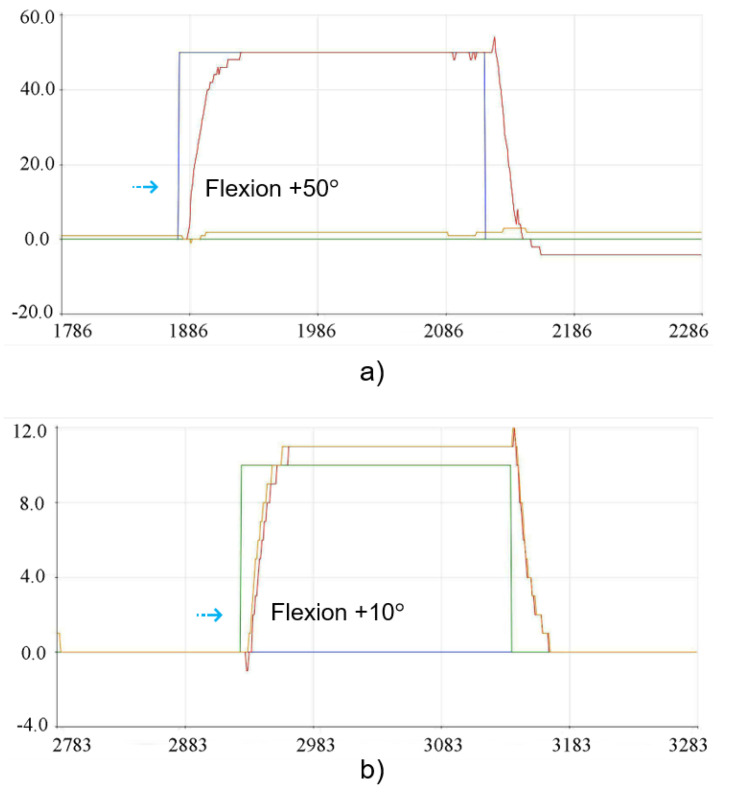
Experimental results for the flexion position: (**a**) Flexion movement +50° for G1. (**b**) Flexion movement +10° for G2.

**Figure 17:**
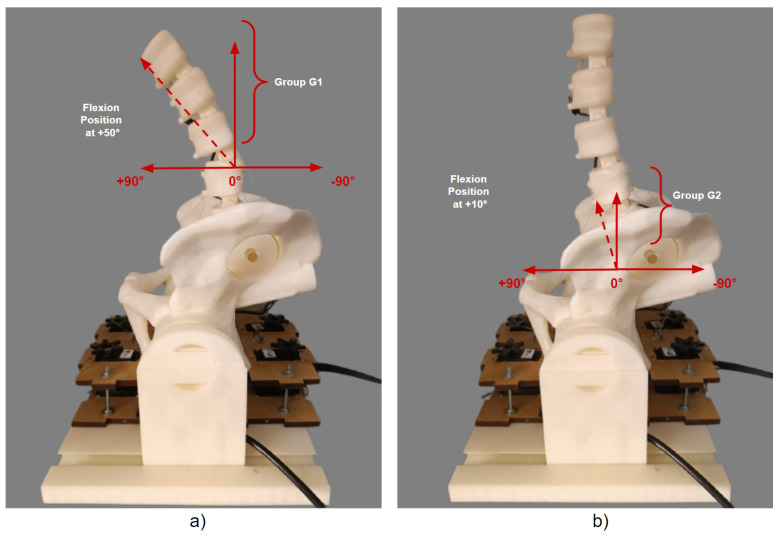
Individual physical results of flexion position for G1 and G2: (**a**) Individual movement of +50° for G1. (**b**) Individual movement of +10° for G2.

Considering the flexion position, the angles determined for each test group comprised +50° for G1 and +10° for G2, and the implementation of the execution tests is shown in the graph images in Figure 16. The results of the positioning of the individual physical tests for each group can be seen in Figure 17.

**Figure 18:**
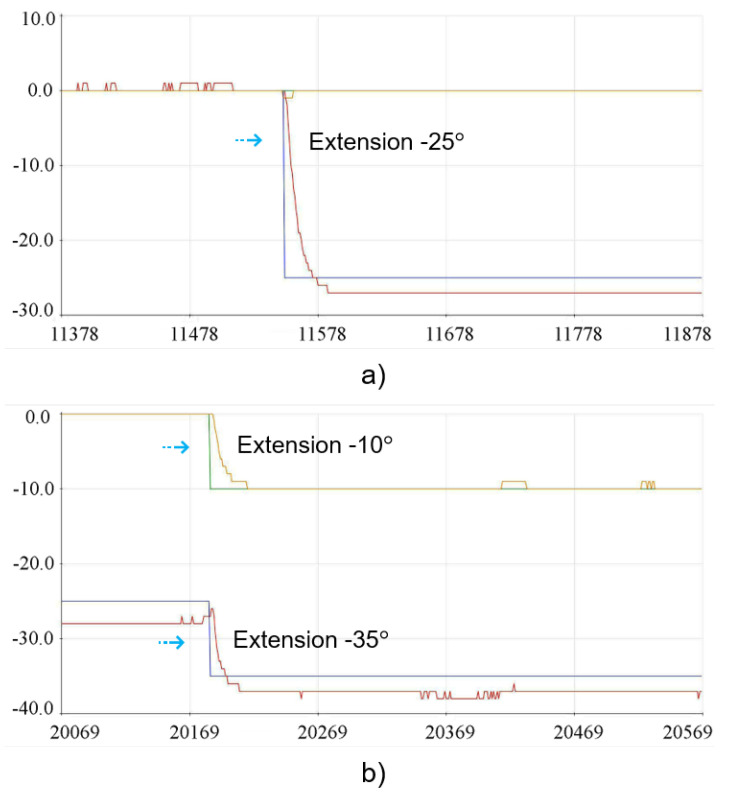
Implementation results for the joint extension position: (**a**) Extension movement −25° for G1. (**b**) Extension movement −10° for G2.

**Figure 19:**
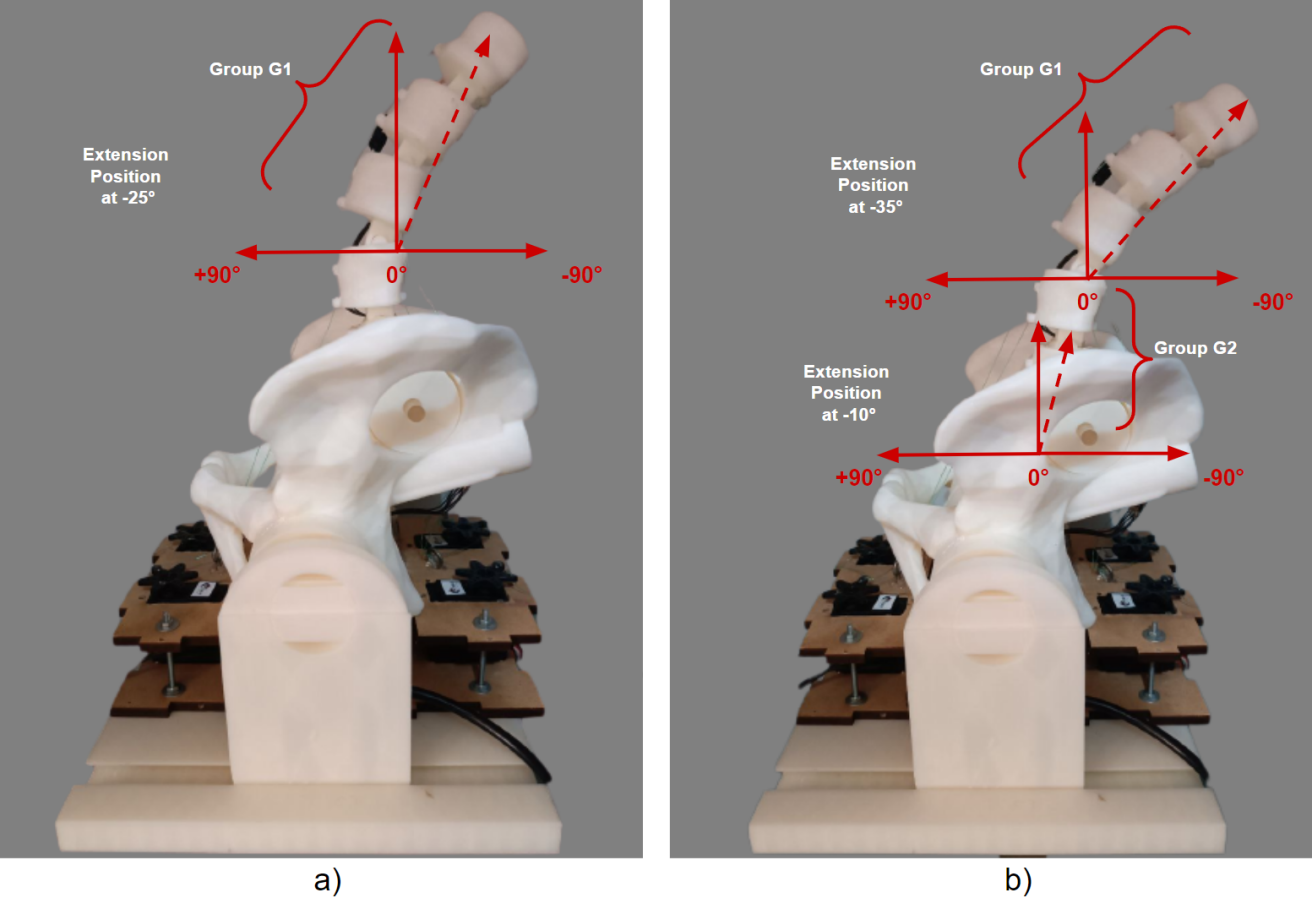
Joint physical results of the extension position for G1 and G2: (**a**) Movement together from −25° to G1. (**b**) Movement together from −10° to G2.

The implementation tests shown in the graphs in Figure 18 were started by indicating −25° for G1 and then −10° for G2 with the complete extension of −35° for G1, taking into account the 10° added to the displacement reached by G2. The physical visualization of the 3D model’s extension positioning can be seen in Figure 19.

**Figure 20:**
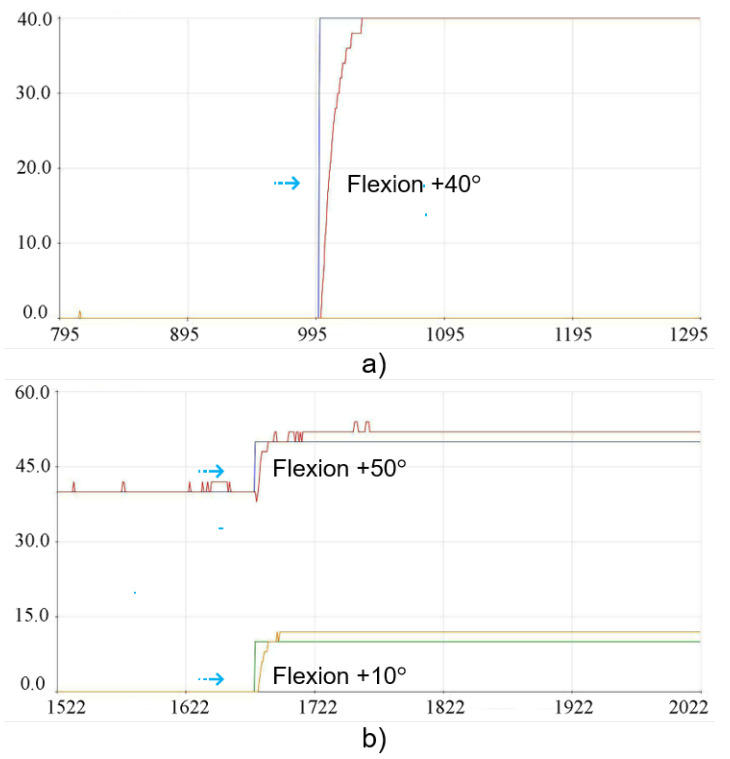
Result of the implementations for joint flexion position: (**a**) Flexion movement +40° for G1. (**b**) Flexion movement +10° for G2.

**Figure 21:**
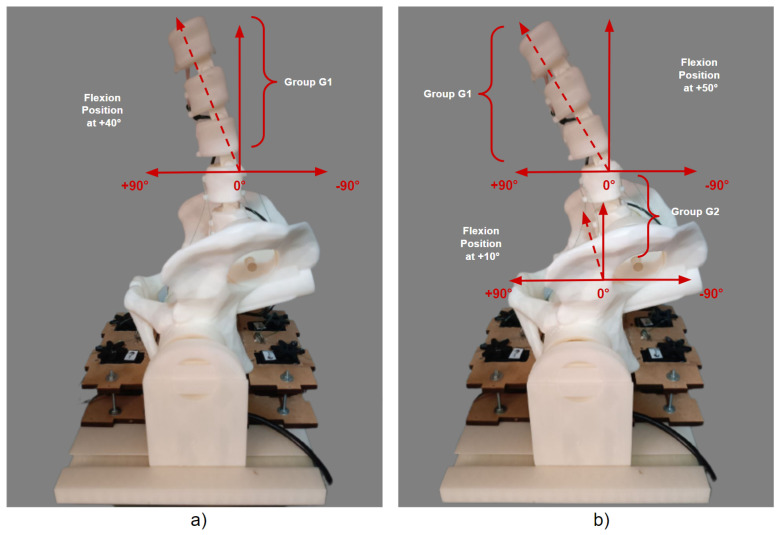
Joint physical results of the flexion position for G1 and G2: (**a**) Movement together +40° for G1. (**b**) Motion together +10° for G2.

The graphs shown in Figure 20 show the execution of the implementation for the tests with +40° for G1 and +10° for G2. The result of the physical positioning after the movement can be seen in Figure 21, where group G1 consequently had 10° added due to the displacement carried out jointly with group G2, totaling +50° of flexion.

## 5. Discussion

In the first stage of the tests, the movements of −5° extension for G1 and +4° flexion for G2 were indicated. Next, −35° of extension for G1 and −10° of extension for G2 were tested. Later, movements of +50° flexion for G1 and +10° flexion for G2 were indicated. These first tests were carried out separately for each movement indicated for the G1 and G2 groups. The second stage of the tests was carried out sequentially with the main aim of positioning and maintaining the G1 group in a specific position and then indicating a position for the G2 group, providing complete displacement of the two groups. In view of this, the movements carried out together with the two groups, G1 and G2, allowed a more accurate observation of the response of the reference positions indicated for the fuzzy controller and the result of the physical implementation of the 3D model. In this sense, in the second stage of the tests, the movements of −25° of extension for G1 and −10° for G2 were indicated, considering the permanence of the position of the G1 group; when moving the G2 group, the G1 group reached −35° of extension. Subsequently, the movements of +40° flexion for G1 and +10° flexion for G2 were indicated, following the same criteria as the previous test with the movement of G1 being retained; by moving group G2, group G1 reached +50° flexion.

The results presented show the general responses of the outputs of the controlled movements. Bearing in mind that only one Arduino Mega microcontroller was used, the intended flexion and extension movements were carried out using a fuzzy controller, but in order to enable more precise tracking of the output signals for the G1 and G2 movements, it was considered necessary to use two fuzzy controllers and/or more precise sensors. The answers presented in the graphs and images show that the model proposed for movement control returns promising results, indicating the ability to control the movement of the 3D model of the lumbar spine.

## 6. Conclusions

This paper presents the development and implementation of a fuzzy controller for the movement of a 3D model of the lumbar spine. The mathematical modeling proposed for simulating the spine model for the controlled environment was based on the Lagrange methodology. The fuzzy controller considered position, velocity and acceleration as variables to generate the torque signal at the controller’s output. To carry out the physical tests with the 3D model, characterizing a real performance verification environment, where the dynamic simulation model for the control was replaced by the 3D structure of the lumbar spine and the fuzzy controller was in direct execution with the robotic 3D structure, the movements were planned to be carried out by means of tendons driven by servomotors. The five vertebrae of the 3D lumbar spine model were structured into group G1 (L1, L2, L3) and group G2 (L4, L5). The physical experiment tests were carried out using four servomotors, two sensors, one Arduino Mega and nylon wires to replicate flexion and extension movements for the G1 and G2 groups. In the movement positions indicated for group G1, movements of +50°, +40° flexion and −5°, −35°, −25° extension were tested. For group G2, movements of +4°, +10° flexion and −10° extension were tested and reproduced.

The results of the physical experiments showed a variation in the responses with some slight deviations, vibrations and a minimum percentage of 10% overshoots and undershoots in the output responses of the graphs with the fuzzy controller. In this sense, the physical responses of the behavior with a greater displacement in relation to its amplitude showed a more relevant performance in some cases. This was especially true for group G1, which may be due to the wires that drive the group of vertebrae causing the movements for smaller amplitudes to require more meticulous manipulation. In turn, this phenomenon is related to the limitation of the physical movement of the servomotors to the drive of the nylon wires, which have a corresponding range of movement from 0° to 180°. In this way, the physical responses of the behavior of the G2 group showed some signal intensities, considering that the vertebrae of the G2 group reach an amplitude of only +10° and −10°, being in a position closer to the pelvis and the servomotors. Considering that the MPU6050 has a typical accuracy of ±3–5% of the measurement range for both the gyroscope and the accelerometer, substitution with other sensors with better accuracy may reduce the permanent response rate errors.

Bearing in mind that only one fuzzy controller was used to drive group G1 and group G2, in addition to the physical conditions related to the electronic devices used and the presence of external forces that oppose the movements directed by the fuzzy controller in the 3D structure, despite this, the responses of the controlled movements are evaluated as promising and relevant, indicating in this case the need to make just a few adjustments to make the output signals of the fuzzy controller more precise.

Future work can be carried out to extend the dynamic model matrix, in relation to the manipulation of the complete mathematical model of the lumbar spine, so that the control system and the dynamic model incorporate the five degrees of freedom, allowing detailed movements of each vertebra to be carried out. Continuously rotating servomotors (360°) can be used to guarantee the best movement for each vertebra in relation to the complete range required for flexion and extension mobility. In addition, electronic devices could be added to the controlled physical system, with the inclusion of sensors and microcontroller boards, so that new fuzzy controllers can be inserted into the developed system.

## Figures and Tables

**Table 1:** First rule base sequence established for the fuzzy controller.

Position 1	Position 2	Speed 1	Speed 2	Acceleration 1	Acceleration 2	Torque 1	Torque 2
N5	N2	LVF	LVF	-	-	L4	L3
N5	N2	LF	LF	-	-	L5	L3
N5	N2	S	S	-	-	L5	L3
N5	N2	RF	RF	-	-	L5	L3
N5	N2	RVF	RVF	-	-	L5	L3
N5	N5	LVF	LVF	-	-	L4	L4
N5	N5	LF	LF	-	-	L5	L5
N5	N5	S	S	-	-	L5	L5
N5	N5	RF	RF	-	-	L5	L5
N5	N5	RVF	RVF	-	-	L5	L5
N4	N4	LVF	LVF	-	-	L3	L3
N4	N4	LF	LF	-	-	L4	L4
N4	N4	S	S	-	-	L4	L4
N4	N4	RF	RF	-	-	L4	L4
N4	N4	RVF	RVF	-	-	L5	L5
N3	N3	LVF	LVF	-	-	L2	L2
N3	N3	LF	LF	-	-	L4	L4
N3	N3	S	S	-	-	L4	L4
N3	N3	RF	RF	-	-	L4	L4
N3	N3	RVF	RVF	-	-	L4	L4
N2	N2	LVF	LVF	-	-	L3	L3
N2	N2	LF	LF	-	-	L3	L3
N2	N2	S	S	-	-	L3	L3
N2	N2	RF	RF	-	-	L4	L3
N2	N2	RVF	RVF	-	-	L4	L3
N1	P1	LVF	LVF	-	-	L2	R3
N1	P1	LF	LF	-	-	L2	R4
N1	P1	S	S	-	-	L5	R3
N1	P1	RF	RF	-	-	L4	R1
N1	P1	RVF	RVF	-	-	L3	R1
N1	N1	-	-	P	P	L1	L1
N1	N1	LVF	LVF	-	-	L2	L2
N1	N1	LF	LF	-	-	L2	L2
N1	N1	S	S	-	-	L5	L3
N1	N1	RF	RF	-	-	L4	L4
N1	N1	RVF	RVF	-	-	L3	L3
Z	Z	-	-	Z	Z	Z	Z
Z	Z	LVF	LVF	-	-	Z	Z
Z	Z	LF	LF	-	-	Z	Z
Z	Z	S	S	-	-	Z	Z
Z	Z	RF	RF	-	-	Z	Z
Z	Z	RVF	RVF	-	-	Z	Z

**Table 2:** Second sequence of the rule base established for the fuzzy controller.

Position 1	Position 2	Speed 1	Speed 2	Acceleration 1	Acceleration 2	Torque 1	Torque 2
P1	P1	LVF	LVF	-	-	R3	R3
P1	P1	LF	LF	-	-	R4	R4
P1	P1	S	S	-	-	R5	R3
P1	P1	RF	RF	-	-	R1	R1
P1	P1	RVF	RVF	-	-	R1	R1
P1	P1	-	-	N	N	R1	R1
P2	P2	LVF	LVF	-	-	R4	R3
P2	P2	LF	LF	-	-	R4	R3
P2	P2	S	S	-	-	R3	R3
P2	P2	RF	RF	-	-	R3	R3
P2	P2	RVF	RVF	-	-	R3	R3
P3	P3	LVF	LVF	-	-	R4	R4
P3	P3	LF	LF	-	-	R4	R4
P3	P3	S	S	-	-	R4	R4
P3	P3	RF	RF	-	-	R4	R4
P3	P3	RVF	RVF	-	-	R4	R4
P4	P4	LVF	LVF	-	-	R5	R5
P4	P4	LF	LF	-	-	R5	R5
P4	P4	S	S	-	-	R5	R5
P4	P4	RF	RF	-	-	R5	R5
P4	P4	RVF	RVF	-	-	R2	R2
P5	P5	LVF	LVF	-	-	R6	R6
P5	P5	LF	LF	-	-	R5	R5
P5	P5	S	S	-	-	R5	R5
P5	P5	RF	RF	-	-	R5	R5
P5	P5	RVF	RVF	-	-	R4	R4
P6	P6	LVF	LVF	-	-	R7	R7
P6	P6	LF	LF	-	-	R6	R6
P6	P6	S	S	-	-	R6	R6
P6	P6	RF	RF	-	-	R6	R6
P6	P6	RVF	RVF	-	-	R5	R5
P7	P2	LVF	LVF	-	-	R7	R3
P7	P2	LF	LF	-	-	R7	R3
P7	P2	S	S	-	-	R7	R3
P7	P2	RF	RF	-	-	R7	R3
P7	P2	RVF	RVF	-	-	R6	R3
P7	P7	LVF	LVF	-	-	R7	R7
P7	P7	LF	LF	-	-	R7	R7
P7	P7	S	S	-	-	R7	R7
P7	P7	RF	RF	-	-	R7	R7
P7	P7	RVF	RVF	-	-	R6	R6

## Data Availability

Data are contained within the article.

## References

[1] Karadogan E., Williams R.L. (2013). The robotic lumbar spine: Dynamics and feedback linearization control. Comput. Math. Methods Med..

[2] Garg B., Mehta N. (2018). Current status of 3D printing in spine surgery. J. Clin. Orthop. Trauma.

[3] Triolo R.J., Bailey S.N., Miller M.E., Lombardo L.M., Audu M.L. (2013). Effects of stimulating hip and trunk muscles on seated stability, posture, and reach after spinal cord injury. Arch. Phys. Med. Rehabil..

[4] Swain C.T., Pan F., Owen P.J., Schmidt H., Belavy D.L. (2020). No consensus on causality of spine postures or physical exposure and low back pain: A systematic review of systematic reviews. J. Biomech..

[5] Eremina G., Smolin A., Martyshina I. (2022). Convergence analysis and validation of a discrete element model of the human lumbar spine. Rep. Mech. Eng..

[6] Turbucz M., Pokorni A.J., Szőke G., Hoffer Z., Kiss R.M., Lazary A., Eltes P.E. (2022). Development and Validation of Two Intact Lumbar Spine Finite Element Models for In Silico Investigations: Comparison of the Bone Modelling Approaches. Appl. Sci..

[7] Kahraman C., Deveci M., Boltürk E., Türk S. (2020). Fuzzy controlled humanoid robots: A literature review. Robot. Auton. Syst..

[8] El-Khatib M.F., Maged S.A. (2021). Low level position control for 4-DOF arm robot using fuzzy logic controller and 2-DOF PID controller. Proceedings of the 2021 International Mobile, Intelligent, and Ubiquitous Computing Conference (MIUCC).

[9] Angst L.R. (2022). Construção e Validação de um Modelo Experimental da Cinesiologia da Coluna Lombar Humana. Master’s Thesis.

[10] Paixão T., Alvarez A.B., Florez R., Palomino-Quispe F. (2023). Motion control of a robotic lumbar spine model. Proceedings of the International Work-Conference on Bioinformatics and Biomedical Engineering.

[11] Paixão T., Alvarez A.B., Florez R., Palomino-Quispe F., Angst L., Maggi L. (2023). Development of Simplified Lumbar Spine Mechanism Implemented with Tendon-Driven Motion. Proceedings of the 2023 27th International Conference on Methods and Models in Automation and Robotics (MMAR).

[12] Kakehashi Y., Okada K., Inaba M. (2020). Development of Continuum Spine Mechanism for Humanoid Robot: Biomimetic Supple and Curvilinear Spine Driven by Tendon. Proceedings of the 2020 3rd IEEE International Conference on Soft Robotics (RoboSoft).

[13] Clifton W., Nottmeier E., Damon A., Dove C., Chen S.G., Pichelmann M. (2019). A feasibility study for the production of three-dimensional-printed spine models using simultaneously extruded thermoplastic polymers. Cureus.

[14] Bohl M.A., McBryan S., Newcomb A.G., Lehrman J.N., Kelly B.P., Nakaji P., Chang S.W., Uribe J.S., Turner J.D., Kakarla U.K. (2020). Range of motion testing of a novel 3D-printed synthetic spine model. Glob. Spine J..

[15] Bohl M.A., Morgan C.D., Mooney M.A., Repp G.J., Lehrman J.N., Kelly B.P., Chang S.W., Turner J.D., Kakarla U.K. (2019). Biomechanical testing of a 3D-printed L5 vertebral body model. Cureus.

[16] DiAngelo D., Hoyer D., Chung C. (2019). Biomechanical evaluation of a full-length (T12-S) synthetic lumbar spine model. MOJ Appl. Bionics Biomech..

[17] Li C., Yang M., Xie Y., Chen Z., Wang C., Bai Y., Zhu X., Li M. (2015). Application of the polystyrene model made by 3-D printing rapid prototyping technology for operation planning in revision lumbar discectomy. J. Orthop. Sci..

[18] Wilcox B., Mobbs R.J., Wu A.M., Phan K. (2017). Systematic review of 3D printing in spinal surgery: The current state of play. J. Spine Surg..

[19] Cho W., Job A.V., Chen J., Baek J.H. (2018). A review of current clinical applications of three-dimensional printing in spine surgery. Asian Spine J..

[20] Urrea C., Kern J., Alvarado J. (2020). Design and evaluation of a new fuzzy control algorithm applied to a manipulator robot. Appl. Sci..

[21] Bikova M., Latkoska V.O., Hristov B., Stavrov D. (2022). Path Planning Using Fuzzy Logic Control of a 2-DOF Robotic Arm. Proceedings of the 2022 IEEE 17th International Conference on Control & Automation (ICCA).

[22] Hey H.W.D., Tan K.A., Thadani V.N., Liu G.K.P., Wong H.K. (2020). Characterization of sagittal spine alignment with reference to the gravity line and vertebral slopes: An analysis of different roussouly curves. Spine.

[23] Mitchell T., O’Sullivan P.B., Burnett A.F., Straker L., Smith A. (2008). Regional differences in lumbar spinal posture and the influence of low back pain. BMC Musculoskelet. Disord..

